# Bioremediation of chlorpyrifos residues using some indigenous species of bacteria and fungi in wastewater

**DOI:** 10.1007/s10661-023-11341-3

**Published:** 2023-05-31

**Authors:** Eman Mohammad Elzakey, Sabha Mahmoud El-Sabbagh, Eman El-Sayed Nour Eldeen, Ibrahim Abdel-Aziz Adss, Atef Mohamed Khedr Nassar

**Affiliations:** 1 Department of Microbiology, Faculty of Science, Menofia University, Shibin El Kom, Menoufia Egypt; 2 Plant Protection Department (Pesticides), Faculty of Agriculture, Damanhour University, Damanhour, El-Beheira Egypt; 3 Department of Plant Pathology (Genetics), Faculty of Agriculture, Damanhour University, Damanhour, El-Beheira Egypt

**Keywords:** Bioremediation, Organophosphate insecticides, Wastewater, Bacteria, Fungi

## Abstract

Pollutants cause a huge problem for humans, animals, plants, and various ecosystems, especially water resources. Agricultural, domestic, and industrial waste effluents change the water quality and affect living microorganisms. Therefore, the current study aimed to identify possible microorganisms in wastewater as potential bioremediation agents of pesticide residues. Wastewater samples were collected from El-Khairy agricultural drainage, which receives agricultural and domestic wastes. Bacteria and fungi species were isolated as clean cultures. Wastewater samples were analyzed for pesticide residues via gas chromatography-mass spectroscopy (GC–MS) system. Results uncovered the presence of ten pesticides ranging from 0.0817 to 28.162 µg/l, and the predominant pesticide was chlorpyrifos. Along with that, about nine species (3 bacterial and 6 fungal) were relatively efficient in the removal of chlorpyrifos residues up to 2000 µg/l with removal percentages ranging from 24.16 to 80.93% under laboratory conditions. Two bacterial isolates proficiently degraded significant amounts of chlorpyrifos: *Bacillus cereus* strain PC2 (GenBank accession No. MZ314010) and *Streptomyces praecox* strain SP1 (GenBank accession No. MZ314009). In-site bacterial and fungal isolates defined in the current study were proficient in cleaning wastewater of chlorpyrifos pesticide residues.

## Introduction

Water shortage and quality deterioration, along with the ever-increasing population, would be great challenges facing many countries worldwide (Abdel-Gawadh et al., 2004). By 2050, about 6 billion people will suffer from the scarcity of clean water (Boretti & Rosa, 2019; UN-WWDR, 2020). In Egypt, water shortage is an alarming problem; its effects have been growing in recent years. Egypt depends mainly on the Nile River to sustain freshwater supplies (Dakkak, 2020). Since 1959, the Nile provides Egypt with about 55.5 billion m^3^/year; hence, the water share per capita has been reduced from 2560 m^3^/year in 1959 to 980 m^3^/year in 2000 and is expected to reach 637 m^3^/year by 2025 (Ashour et al., 2003).

Along with that, water resources are polluted with industrial, domestic, and agricultural waste (Ashour et al., 2003; Koshal, 1976). For example, heavy metals, asbestos, nitrates, detergents, solvents, fertilizers, and pesticides were listed as major pollutants of the River Nile (El-Sheekh, 2009). Pesticides cause numerous negative health and environmental effects (Nicolopoulou-Stamati et al., 2016). Especially, the long-lasting pesticides in ecosystems, for instance, organochlorine insecticides were found in water resources after more than 20 years of their use (Caughey, 1999; Seo et al., 2007). Specifically, organophosphorus pesticides contaminated various ecosystems around the world and caused adverse effects to millions of people with over 200,000 deaths annually (Abraham et al., 2013).

Furthermore, improper disposal and overuse of pesticides have added large amounts to water and soil environments. Pesticides might reach water through runoff with irrigation water, air drifting, leaching, and/or direct application (dusting and spraying), which in turn affect the water quality and aquatic organisms (Abbassy et al., 2020). Consequently, human exposure to these pesticides’ residues might occur throughout food chain leading to various adverse impacts (Agrawal et al., 2010; Hakeem et al., 2016). Therefore, cleaning water resources through the implementation of proper remediation methods is extremely needed. Specifically, the strategy of biological remediation (bioremediation) is widely employed. Because it depends mainly on the nature and type of pollutants and the metabolic degradation mechanism of microbes (Megharaj et al., 2011; Moss, 2008). Also, its applications are rapidly adapted as a suitable alternative to conventional clean-up technologies (Vidali, 2001), where microorganisms are more adjustable to environmental changes and deterioration (Vroumsia et al., 2005).

Moreover, the bioremediation rate and level of a pesticide depend on its bioavailability, uptake rate by the microbiological cell, and the growth rate of the cells with the pesticide as the energy source (Abatenh et al., 2017; Odukkathil & Vasudevan, 2013). Recently, diverse organisms, including algae, bacteria, and fungi and plants were employed to clean polluted environments of pesticides (Díaz, 2004; Pushpanathan et al., 2014; Vay et al., 2001). Specifically, several bacterial strains showed a degradation potential of organophosphate insecticides (Cycoń et al., 2009). *Pseudomonas fluorescens*, *Brucella melitensis*, *Bacillus subtilis*, and *P. aeruginosa* were able to remove 89, 87, 85, and 92% of chlorpyrifos (CPF), respectively, after 30 days of incubation (Lakshmi et al., 2008). Also, *Bacillus* sp., *Brevundimonas* sp., *Pseudomonas* sp., *Sphingomonas* sp., and *Stenotrophomonas* sp. significantly degraded from 37 to 100 mg/l/d of CPF (Li et al., 2008). The *P. aeruginosa*, *B. cereus*, *Serratia marcescens*, and *Klebsiella* sp. were effective in removing 84, 84, 81, and 80%, respectively, of CPF from liquid media after 20 days, while after 30 days in soils, they removed about 92, 60, 56, and 37%, respectively (Lakshmi et al., 2009). Similarly, *Lactobacillus lactis*, *L. fermentum*, and *E. coli* efficiently converted CPF to its oxon and DETP metabolites (Harishankar et al., 2013).

The utilization of pesticides by microorganisms as sources of minerals (carbon and phosphorous) and energy would help in cleaning various water resources from their residues. Therefore, the current study aimed to isolate indigenous bacteria and fungi and screen their potential as bioremediation agents of wastewater of pesticide residues.

## Materials and methods

### Chemicals

Acetonitrile HPLC-grade and culture media were purchased from local chemical providers. DNA purification kit (Germany) and PCR clean-up kit were from Maxim Biotech Inc. (USA). The internal standard (TPP) and extraction (Cat#5982–0650) and dispersive SPE clean-up (Cat#5982–5056) kits were purchased from Technoscient for Lab & Optical Product, Cairo, Egypt. Certified reference standard materials of pesticides were obtained from ULTRA Scientific Analytical Solutions (RI, USA) (Table 1).

**Table 1:** List of analyzed pesticides in wastewater samples using the GC–MS based on a preliminary survey of applied pesticides on crops around the study locations

Grouping	Common name
Acaricides, insecticides, and nematicides	Azinophos-methyl (20)*, bifenthrin (15), cadusafos (50), carbofuran (15), chlorpyrifos (10), cyhalothrin (10), cypermethrin (15), deltamethrin (10), dimethoate (50), diazinon (20), dichlorvos (10), esfenvalerate (15), malathion (10), oxamyl (20), permethrin (15), pirimiphos-methyl (10), profenofos (15), quinalphos (15), sulfotep (20), tolfenpyrad (20), and thiamethoxam (50)
Fungicides	Chlorothalonil (25), dicloran (20), difenoconazole (20), diniconazole (20), ethofumesate (25), lenacil (20), penconazole (30), and propiconazole (15)
Herbicides	Atrazine (10), Butralin (20), Pendimethalin (50), and Simetryn (25)

^*^Numbers between brackets represent the limits of quantification (LOQ; µg/l). Method quantification limit of the tested pesticide was calculated using the equation LOQ = 10σ/S, where σ is the standard deviation of the *y*-intercept and *S* is the slope of the corresponding calibration curve

### Source of water samples

**Fig. 1:**
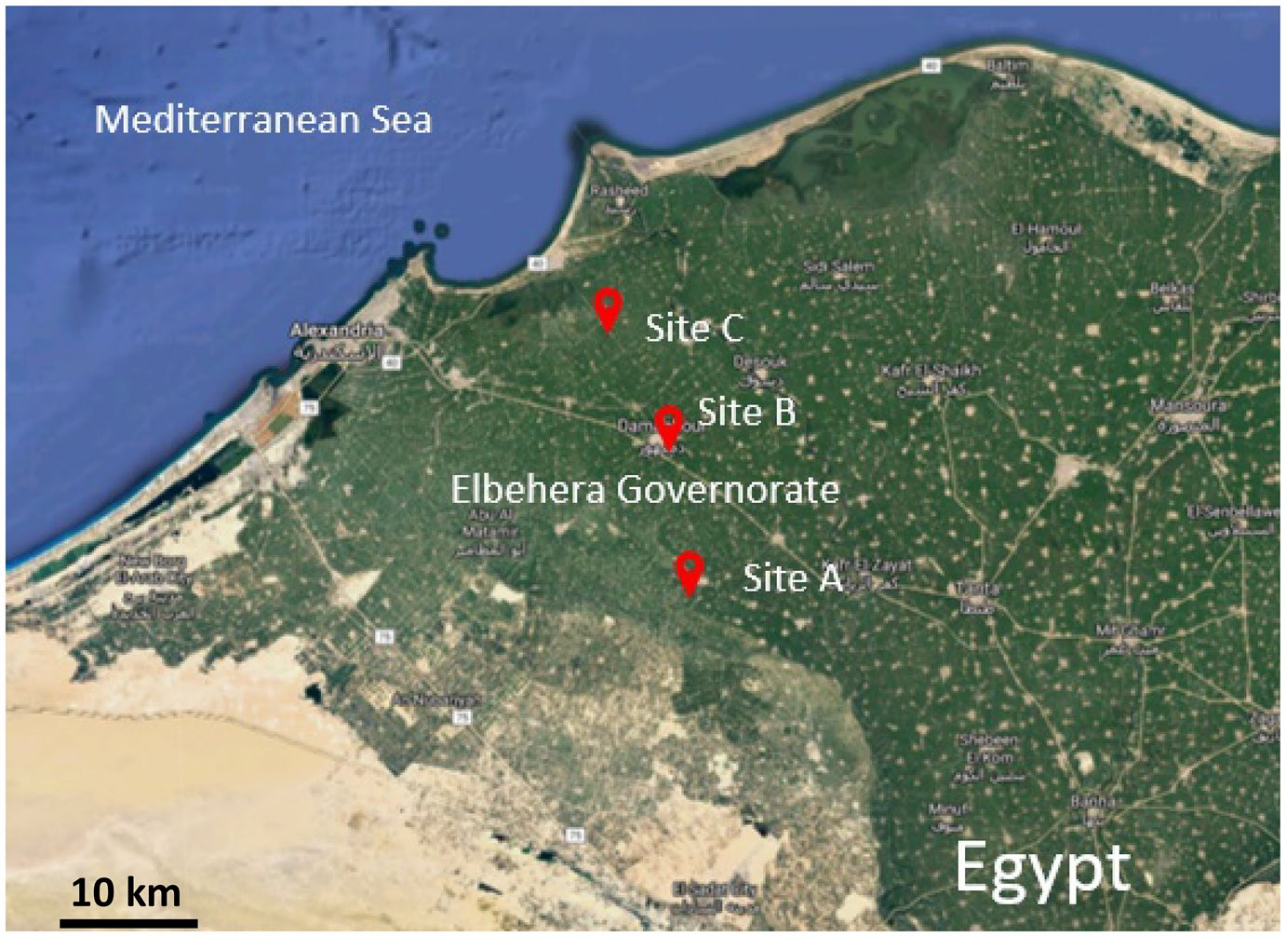
Map illustrating the study location and sample collection sites North of Egypt

Wastewater samples were collected from three locations: start, middle, and end at the El-Khairy drainage, El-Beheira Governorate, north of Egypt (Fig. 1). The drainage receives industrial, domestic, and agricultural effluents. Its water is being re-used for the irrigation of vast areas of agricultural lands; the total served area is 27,500 feddan. It is considered the main agricultural drainage of a length of 21.65 km and receives an average discharge of 14.2 m^3^/s (Veeningen, 1999).

### Sampling of wastewater

Subsurface water samples (25 cm below the surface) were collected by water sampler into cleaned and sterilized one-liter Pyrex borosilicate dark glass bottles (Radwan et al., 2019). Water bottles were used for the bacteria and fungi screening and isolation and pesticide residue analyses. Samples were transferred to the Pesticides Residues Analysis and Toxicity Laboratory (PRATL), Faculty of Agriculture, Damanhour University, for analysis within a few hours of collection.

### Isolation of microorganisms from wastewater

Microorganisms found in wastewater samples were separated and sub-cultured on potato dextrose agar for fungi isolation (200 g of infusion from potatoes, 20 g of dextrose, 15 g of agar at pH 5.6 ± 0.2) and plate count agar for isolation of the bacteria colonies (5 g of tryptone, 2.5 g of yeast extract, 1 g glucose, and 15 g agar at pH 7.0 ± 0.25) at 25 °C. Nine samples were collected; 3 from each collection point as replicates and 3 plates per replicate were planted from each sample under sterilized conditions and incubated at 37 °C. The complete growth of the microbe was reached after about 7 days of incubation. Each microorganism was transferred into a new Petri dish. The subculture of each microbe was repeated several times until a visually clean culture was obtained.

### Analysis of pesticide residues in wastewater using GC–MS

#### Pesticide extraction and clean-up

Pesticide residues in wastewater were extracted and cleaned up using a modified method of Anastassiades (Anastassiades et al., 2003). Extraction (Cat#5982–0650) and dispersive SPE clean-up (Cat#5982–5056) kits of Agilent Technologies were used. Specifically, about 10 ml of wastewater was vortexed with 10 ml of 0.1% acidified MeCN for 1 min. Then 4 g and 1 g of MgSO_4_ (anhy) and NaCl, respectively, were thoroughly mixed for 1 min. Then the internal standard triphenyl phosphate (TPP) solution was added to tubes and shaken for 30 s. Then tubes were centrifuged at 1350 × *g* for 10 min (Hermle Labortechnik GmbH, Siemensstr 25 D-78564 Wehingen, Germany). About 1 ml of supernatant (acetonitrile) was mixed by hand for 5 min with 25 mg PSA sorbent and 150 mg MgSO_4_ (anhy) and centrifuged for 5 min at 1350 × *g*. About 500 µl of each tube was filtered through 0.22-μm PTFE filters (Millipore, USA) into HPLC vials for GC–MS analysis.

#### Separation and determination conditions

Separation and identification of residues in wastewater samples and recoveries were accomplished using an Agilent GC–MS system in split-less mode. Exactly, 2 µl of each sample was injected into an HP-5MS capillary column (30 m × 0.53 mm i.d. 0.25 µm film thickness). Separation conditions were as reported by AOAC (AOAC, 2007), where the initial temperature of the column was set at 80 °C for 6 min, increased at 15 °C/min to 215 °C (held for 1 min), and then the column was heated to 230 °C at 5 °C/min and to 290 °C at 5 °C/min (held for 2 min). The carrier gas was the helium gas at a constant flow rate of 1.1 ml/min. The observed pesticides were identified by the full mass spectra scans using the total ion chromatogram (TIC) and search of spectra in the EI-MS libraries. Concentrations of identified pesticides were calculated based on a standard curve of each compound.

#### Quality control parameters

The intra-day assay (repeatability) and inter-day assay (intermediate precision) of the used analytical technique were calculated according to Ermer (2005). Also, the precision expressed as coefficients of variation, limits of detection (LOD), and quantification (LOQs) were estimated. For recovery studies, water samples were fortified with 0.1 and 1 µg/l of each standard and analyzed following the above-mentioned methods. Then percentages of recovery samples were reported ± relative standard deviation (%RSD) (Frenich et al., 2006). All detected amounts of pesticides residues were corrected considering the recovery percentages (Urovic & Orevi, 2011).

#### Microorganisms’ tolerance assay

The radial growth of separated and identified fungi and bacteria on media mixed with the prevalent insecticide in wastewater, chlorpyrifos (CPF), was examined. Five concentrations of CPF (0, 100, 500, 1000, and 2000 µg/l) were prepared in the growing media. Five plates of each microorganism were used as replicates per each concentration. Microorganisms were incubated with the CPF at 37 °C for 1 week, and then their radial growth was photographed and recorded. Then competence of growth fungi and bacteria on such media with the insecticide was calculated compared to control plates. This experiment was repeated six times. Then the performance of the potential bioremediation activity of organisms was examined using 500, 1000, and 2000 µg/l. The residues of CPF in media after the incubation time were measured using the GC–MS as described previously.

#### Identification of fungal isolates

The fungal isolates were cultured onto clean growth media until pure cultures were obtained and used for various evaluations and identification (Lichtwardt, 1985). Microscopic observations were done on mounted cultures using lactic acid. The morphological traits of each fungal colony were observed and recorded as described in fungal atlases (Klich, 1990).

#### Identification of bacterial isolates

Taxonomic characterization of isolated bacteria was conducted at the genus level based on morphological, physiological, and biochemical traits and following the method reported in the manuals of Bergey of Systematic Bacteriology (James, 1998) and Cowan and Steel’s (Barrow & Feltham, 1993).

### Molecular characterization of bacterial isolates

#### Extraction of genomic DNA

Two bacterial isolates were chosen to be identified genetically because of their elevated potential in the degradation of CPF insecticide. The DNA of isolated bacteria was extracted using Qiagen DNA kit (Qiagen, Hilden, Germany) following the manufacturer’s guidelines.

#### Amplification of the 16S rRNA

About 350 bp of 16 s rRNA gene was amplified. The 350F and 350R primers that correspond to the 16S rRNA conserved gene sequence of *E. coli*, forward, 5ʹ AGG ACG TGC TCC AAC CGC A ʹ3, and reverse, 5ʹ AAC TGG AGG AAG GTG GGG AT ʹ3 (Sambrook & Russell, 2001). The PCR reaction was as the following: an initial cycle of 95 °C for 5 min and 34 cycles of 95 °C for 1 min, 47 °C for 1 min, and 72 °C for 1 min and then an extension cycle at 72 °C for 10 min. Then amplified products were visualized on 1% agarose gel, stained by ethidium bromide, and photographed using a gel documentation system. Then the purification of amplified PCR products was done using PCR clean-up column kit (Maxim Biotech INC, USA).

### Sequencing and alignment

The 16S gene DNA sequence (excised and purified bands) was performed by Macrogen Company (Seoul, South Korea). The 16S rRNA gene nucleotide sequences of isolated bacterial strains were submitted to the GenBank database under accession numbers MZ314009 and MZ314010.

### Sequence alignment and phylogenetic analysis

Pair-wise and multiple DNA sequence alignments were performed using CLUSTALW program version 1.82 (http://www.ebi.ac.uk/clustalw) (Thompson et al., 1994). Neighbor-joining trees were created using MEGA version 6 (Tamura et al., 2013) from the CLUSTALW for each strain. Comparison between obtained DNA sequence alignments and sequences in GenBank database was completed using BLASTN searches at http://ncbi.nlm.nih.gov.

### Statistical analysis

Experimental data were statistically analyzed using the SAS software (SAS, Cary, USA, version 9.3). The pesticide residues and microbial isolates’ performance and growth were expressed as mean ± SD. Significant means were contrasted using Tukey’s honest significant difference test (HSD) (*P* ≤ 0.05) (SAS, 2013).

## Results

### Pesticide residues in wastewater

#### Quality control limits

The analytical method used in the analysis of pesticides was accurate and suitable based on obtained values of repeatability and intermediate precision (Ermer, 2005). Results of the intra- and inter-assay ranged from 3.47–6.18% and 6.51–10.11%, respectively, for detected pesticides (Table 2). These results were within the acceptable range set by residue analysis laboratories. Also, recovery results of detected pesticides at 0.5 and 5 µg/l levels ranged from 87.65–93.57% and 91.73–95.37%, respectively.

**Table 2:** The mean amounts of pesticide residues and coefficients of variation (CV%) of wastewater samples collected from the three sites at El-Khairy drainage (A: start, B: middle, and C: end) and recovery (%) percentages ± RSD of pesticides from spiked blank water samples using GC–MS

Site	Rt (min)	Pesticide	Amount (µg/L)	CV%		Recovery (%) ± SEM	
				Intra-assay	Inter-assay	0.5 (µg/L)	5 (µg/L)
A	6.565	Lenacil	0.7214	4.56	7.28	89.42 ± 4.18	91.73 ± 5.72
	13.558	Chlorpyrifos	28.162	3.47	6.51	93.57 ± 3.67	93.78 ± 4.87
	15.869	Cypermethrin	4.1404	4.67	8.57	87.65 ± 6.14	94.06 ± 5.71
	16.243	Bifenthrin	0.0517	4.18	9.14	93.26 ± 5.21	95.37 ± 5.08
	17.274	Carbofuran	7.8813	5.16	8.03	91.64 ± 3.67	93.45 ± 4.19
	20.197	Permethrin	0.2081	3.81	6.72	93.46 ± 4.12	95.08 ± 4.61
B	13.588	Chlorpyrifos	16.049	3.47	6.51	93.57 ± 3.67	93.78 ± 4.87
	15.472	Cypermethrin	3.2407	4.67	8.57	87.65 ± 6.14	94.06 ± 5.71
	16.228	Bifenthrin	0.0912	4.18	9.14	93.26 ± 5.21	95.37 ± 5.08
	18.509	Tolfenpyrad	1.0241	5.76	8.05	91.06 ± 6.08	93.28 ± 5.28
	20.149	Permethrin	0.0927	3.81	6.72	93.46 ± 4.12	95.08 ± 4.61
C	5.866	Oxamyl	5.0731	7.81	10.11	92.59 ± 3.64	95.03 ± 3.12
	9.317	Dicloran	0.0817	5.04	9.54	89.71 ± 4.05	91.24 ± 3.56
	9.857	Simetryn	2.3045	4.91	8.63	92.61 ± 3.84	93.26 ± 3.81
	10.529	Sulfotep	1.0251	5.06	7.55	88.64 ± 4.05	89.17 ± 4.23
	13.308	Chlorpyrifos	23.087	3.47	6.51	93.57 ± 3.67	93.78 ± 4.87
	15.648	Cypermethrin	2.3408	4.67	8.57	87.65 ± 6.14	94.06 ± 5.71
	20.465	Ethofumesate	0.0981	6.18	9.07	88.43 ± 5.09	90.13 ± 4.64

^*^Inter-assay and intra-assay precision results were calculated from the analyzed concentrations of each standard pesticide in fortified laboratory blank water samples

#### Pesticide residues in wastewater samples

Analysis of pesticides residues revealed the presence of lenacil, chlorpyrifos, cypermethrin, bifenthrin, carbofuran, and permethrin at 0.721, 28.16, 4.14, 0.052, 7.881, and 0.208 µg/l, respectively, in samples collected from site A (Table 2). Chlorpyrifos, cypermethrin, bifenthrin, tolfenpyrad, and permethrin were found in samples from site B with concentrations of 16.05, 3.24, 0.09, 1.02, and 0.09 µg/l, respectively. Samples from site C had oxamyl, dicloran, simetryn, sulfotep, chlorpyrifos, cypermethrin, and ethofumesate at 5.071, 0.0817, 2.3045, 1.0251, 23.087, 2.3408, and 0.0981 µg/l, respectively. Chlorpyrifos insecticide was the dominant compound in the wastewater samples. Therefore, it was selected for the bioremediation examinations.

#### Microorganisms and chlorpyrifos removal

**Fig. 2:**
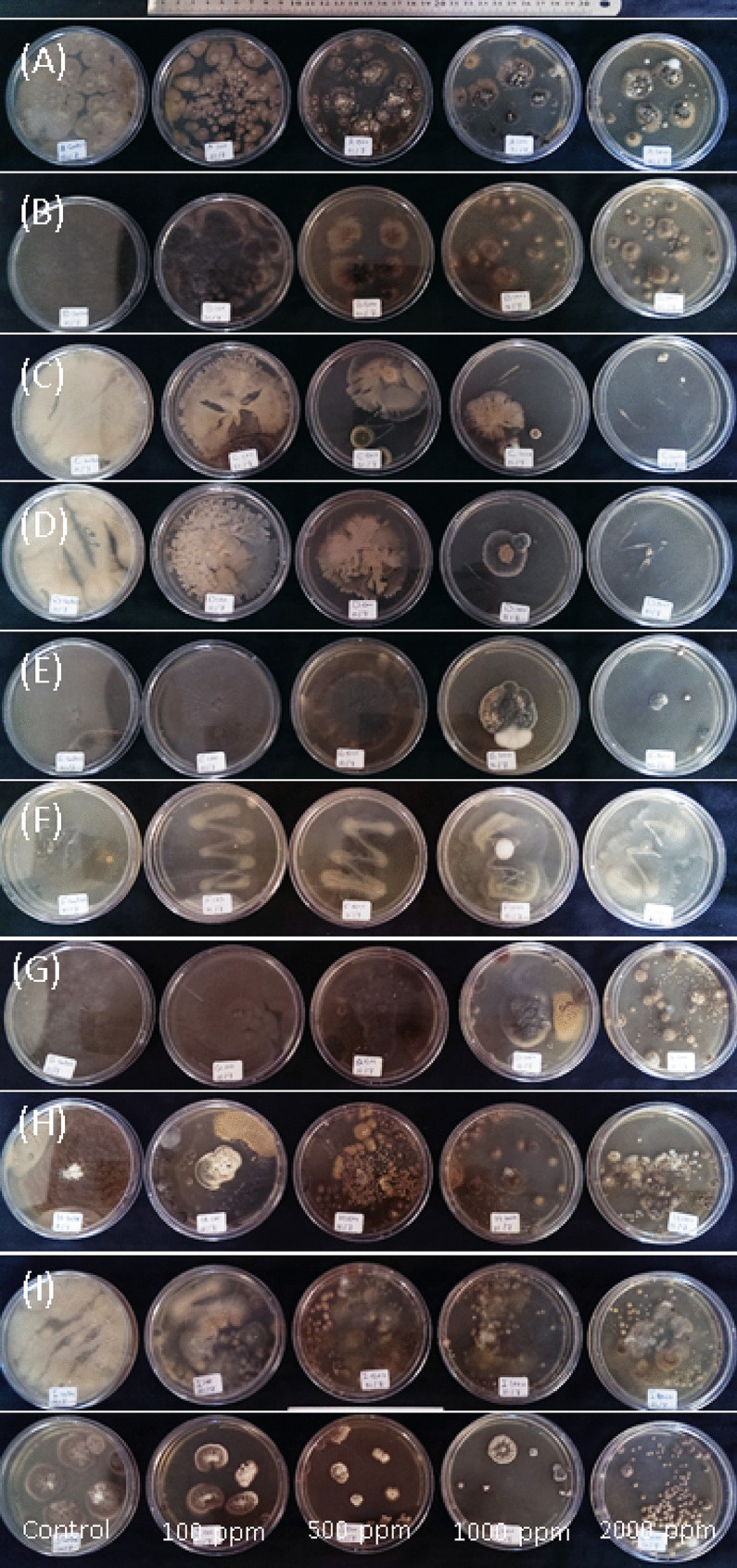
Pictogram illustrates the growth patterns of A, B, D, E, F, G, H, and I isolates after 15 days on media with different concentrations of chlorpyrifos (0, 100, 500, 1000, 2000 mg/l) at 30 ± 2 °C

The results of the growth of separated fungi and bacteria on media mixed with the prevalent insecticide in wastewater (CPF) were presented in Table 3. Five concentrations of CPF (0, 100, 500, 1000, and 2000 µg/l) were added to the growing media. Five Petri dishes of each microorganism were used as replicates per each concentration. The growth of isolated organisms was recorded (Table 3) and photographed (Fig. 2) after 1 week of incubation with or without the insecticide. The microorganisms A, D, and F showed the best growth on plates with or without CPF. The F bacteria was the most grown on media with 2000 µg/l followed by A and D up to 1000 µg/l of CPF compared to all isolated bacteria and fungi (Fig. 2 and Table 3).

**Table 3:** Mean ± SE of growth performance (elliptical area mm^2^) of isolated microbial species on PDA media containing increasing concentrations of chlorpyrifos insecticide (mg/L) after 7 days of incubation at 37° C

Code	Elliptical area (mm^2^)									
	0	± SE	100	± SE	500	± SE	1000	± SE	2000	± SE
Bacterial isolates										
D	200.75^a^	8.99	150.87^b^	7.34	122.46^bc^	8.99	79.02^c^	7.34	18.99^d^	8.99
F	225.06^ab^	7.34	144.98^b^	8.04	240.21^a^	8.99	200.65^ab^	7.34	168.65^ab^	8.99
I	174.72^a^	8.04	102.49^b^	7.34	64.89^c^	10.38	47.10^c^	8.99	41.87^c^	8.99
Fungalisolates										
A	231.17^a^	8.04	109.25^b^	8.04	130.14^b^	8.99	101.74^b^	8.04	7.85^c^	8.99
B	144.63^a^	8.04	112.26^b^	8.99	49.64^c^	8.99	32.81^ cd^	8.99	22.48^d^	8.99
E	184.54^a^	8.04	136.73^ab^	7.34	98.85^b^	8.99	58.48^c^	7.34	42.20^c^	8.99
G	165.92^a^	8.04	129.46^ab^	8.04	112.93^b^	10.38	73.14^bc^	8.04	5.50^d^	8.99
H	127.17^a^	8.99	84.15^a^	8.04	28.10^b^	8.99	29.89^b^	8.99	15.70^b^	8.99
J	185.64^a^	8.99	117.23^b^	10.38	59.72^c^	8.99	39.46^c^	8.99	25.91^c^	8.99

*N* = 6 Petri plates per replicate, 4 replicates per concentration. Strain represents separated microbial organisms after being purified into a clean culture. Isolate code: organism; represents separated microbial organisms after being purified into a clean culture. A: *Aspergillus terreus*, B: *Aspergillus foetidus* var. *pallidus*, D: *Bacillus cereus*, E: *Aspergillus fumigatus* var. *ellipticus*, F: *Streptomyces praecox*, G: *Aspergillus fumigates*, H: *Penicillium janthinellum*, I: unidentified bacteria, J: *Aspergillus fumigatus* var. *ellipticus*. Superscript letters represent significant variation between concentrations for each organism (significance within rows)

**Fig. 3:**
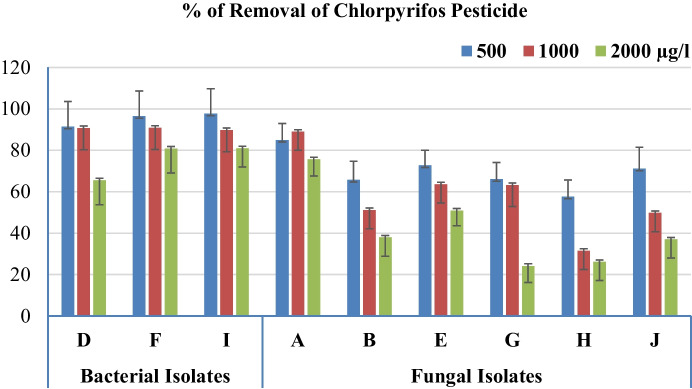
Percentages of removal of chlorpyrifos insecticide by isolated species after 7 days of incubation at 37° C. *N* = 3 Petri plates per replicate, 4 replicates per concentration. Isolated organisms code: A: *Aspergillus terreus*, B: *Aspergillus foetidus* var. *pallidus*, D: *Bacillus cereus*, E: *Aspergillus fumigatus* var. *ellipticus*, F: *Streptomyces praecox*, G: *Aspergillus fumigates*, H: *Penicillium janthinellum*, I: Unidentified bacteria, J: *Aspergillus fumigatus* var. *ellipticus*

Verification of the performance of isolated bacteria (D, F, and I) and fungi (A, B, E, G, H, and J) was examined by challenging their growth on media with 500, 1000, and 2000 µg/l of CPF (Fig. 3). Results showed good potential to use these species in the degradation of CPF up to 1000 µg/l with efficiency (% of removal) ranging from 31.41 (for H) to 90.82% (for F). Specifically, F, I, and A removed about 80.82, 80.93, and 75.59% of the 2000 µg/l of CPF after 1 week.

#### Identification of fungal isolates

**Fig. 4:**
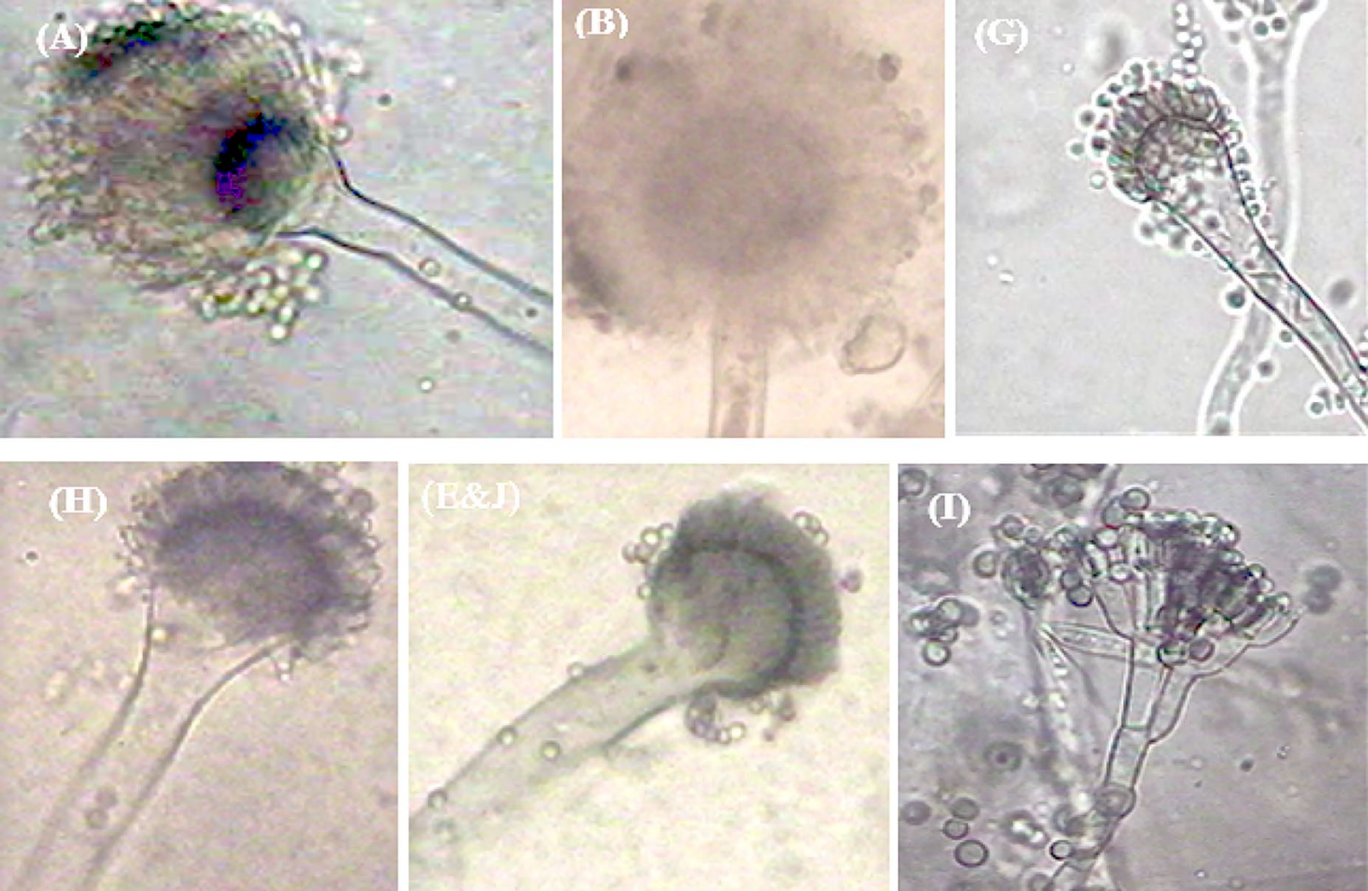
Microscopic pictures of fungal isolates: **A** *Aspergillus terreus*, **B** *Aspergillus foetidus* var. *pallidus*, **G** *Aspergillus fumigatus*, **E&J** *Aspergillus fumigatus* var. *ellipticus*, **H** *Aspergillus flavus*, and **I** *Penicillium janthinellum*

Six different fungal species were identified according to the growth and microscopic characteristics (Table 4 and Fig. 4). Identification was done based on guidelines of the Regional Center for Mycology and Biotechnology (RCMB) using image analysis protocols. The fungal isolates A, B, E and J, and G were *Aspergillus terreus*, *Aspergillus foetidus* var. *pallidus*, *Aspergillus fumigatus* var. *ellipticus*, and *Aspergillus fumigates* according to the database identification program of RCMB For Aspergilli (Klich, 1990). The sixth fungal isolate (H) was *Penicillium janthinellum*; identification was conducted based on current universal keys as described in fungal atlases (James, 1998).

**Table 4:** Fungal identifications according to the protocol of the Regional Center for Mycology and Biotechnology (RCMB) using the image analysis system

Genera (isolate code)	Growth characteristics	Microscopic characteristics
*Aspergillus terreus* (A)	Colonies reach 3.0–3.5 cm diameter on Czapek agar at 25 °C within 7 days with buff to yellow–brown color	CH: columnar, 60.5 μm, VD: sub-globose 15.4 μm in diameter, StP: 6.4×2.3 μm, StS: 6.0×1.2 μm, CD: 5.2 μm in diameter, C: globose, smooth, 2.1 μm in diameter
*Aspergillus foetidus* var. *pallidus* (B)	Colonies reach 5–7 cm diameter in 7 days at 28 °C with velvety, olive black color, reverse first colorless then yellowish brown	CH: black, radiate, VD: globose- sub-globose, 36.0 μm, StP: 13×4.0 μm, StS: 8.0×3.0 μm, CD: 12.0 μm in diameter, C: sub-globose, 4.0 µm
*Aspergillus fumigatus* (G)	Colonies spread rapidly on Czapek agar plates at 25 °C	CH: columnar, 18,030× µm, VD: 25 µm in diameter, fertile over the upper half only, StP: sterigmata in one series 6.0×2.2 µm, CD: 6.0 µm, C: globose, echinulate, green-colored, 2.8 µm in diameter
*Aspergillus fumigatus* var. *ellipticus* (E&J)	Colonies reach 2–3 cm diameter in 7 days at 28 °C on Czapek with blue-green color, reverse colorless	CH: short columnar, VD: flask shape 23.0 μm, StP: 7.9 × 3.0 μm, CD: 12.0 μm in diameter, C: subspherical 3.0 µm
*Penicillium janthinellum* (H)	Colonies on CYA attain 3–4 cm diameter at 25 °C, with white, grayish, buff, pale yellow color, and pale pink mycelium to deep green. Reverse pale yellow, brown or pinkish	CD: 3.5 µm, CD: ellipsoidal 3.0×2.3 µm

*CH* conidial heads, *VD* vesicle diameter, *StP* primary sterigmata, *StS* secondary sterigmata, *CD* conidiophore diameter, *C* conidia diameter

#### Identification of bacterial isolates

The characteristics of the most efficient bacterial isolates (F and I) in degrading CPF were listed in Table 5. The motility, gram staining, sporulation, growth at 40 °C, anaerobic growth, Kovac’s oxidase, gelatin liquefaction, starch hydrolysis, and acid production from lactose, mannose, arabinose, maltose, and sucrose were listed. The isolates were identified as *Streptomyces praecox* (F) and *Bacillus cereus* (I). For further differentiation between the two bacterial strains, similar morphology, physiological, and biochemical traits were reported, except for starch hydrolysis and lactose production that were negative in *B. cereus* and positive in *S. praecox*. Also, *B. cereus* produced acid from sucrose, but *S. praecox* did not.

**Table 5:** Morphological traits and physiological and biochemical activities of *Bacillus cereus* and *Streptomyces praecox* isolated from wastewater samples

Characteristics	Bacterial isolates	
	*Bacillus cereus strain PC2*	*Streptomyces praecox strain SP1*
Cell shape (rods, single)	+	+
Sporulation	+	+
Motility	+	+
Gram reaction	+	+
Catalase activity	+	+
Gelatin liquefaction	+	+
Hydrolysis of starch	-	+
Anaerobic growth	-	-
Kovac’s oxidase	+	+
Growth at 40 °C	+	+
Production of acid from:		
Arabinose	+	+
Lactose	-	+
Mannose	+	+
Maltose	-	-
Sucrose	+	-

#### Phylogenetic identification of bacteria

The 16S rRNA gene sequences of *Bacillus cereus* PC2 (GenBank Acc# MZ314010) and *Streptomyces praecox* SP1 (GenBank Acc# MZ314009) were constructed via molecular identification. Results of DNA sequences of the 16S gene of F and I bacterial isolates were as the following for F, identified as *Streptomyces praecox*, with a sequence of the following:

- GACGGCCTTCGGGTTGTAAACCTCGGGCAGCAGGGAAGAAGCGCAAGTGACGGTACCTGCAGAAGAAGCG
- CCGGCTAACTACGTGCCAGCAGCCGCGGTAATACGTAGGCCCCAAGCGTTGTCCGGAATTATTGGGCGTA
- AAGAGCTCGTAGGCGGCTTGTCACGTCGGATGTGAAAGCCCGGGGCTTAAGGGGGGGTCTGCATTCGATA
- CGGGCTAGCTAGAGTGTGGTAGCCCAGATCGGAATTCCTGGTGTAGCGGTGAAATGCGCAGATATCAGGA
- GGAACACCGGTGGCGTTGGCGGATCTCTGGGCCATTACTGACGCTGAGGAGCGAAAGCGTGGGGAGCGAA
- CAGGATTAGATACCCTGGTAGTCCACGCCGTAAACGTTGGGAACTAGGTGTTGGCGACATTCCACGTCGT
- CGGTGCCGCAGCTAACGCATTAAGTTGGGGGCCTGGGGAGTACGGCCGCAAGGCTAAAACTCAAAGGAAT

The second bacteria (I) was identified as *Bacillus cereus* with a DNA sequence of the following:

- CAGACTCCTACGGGAGGCAGCAGTAGGGAATCTTCCGCAATGGACGAAAGTCTGACGGAGCAACGCCGCG
- TGAGTGATGAAGGCTTTCGGGTCGTAAAACTCTGTTGTTAGGGAAGAACAAGTGCTAGTTGAATAAGCTG
- GCACCTTGACGGTACCTAACCAGAAAGCCACGGCTAACTACGTGCCAGCAGCCGCGGTAATACGTAGGTG
- GCAAGCGTTATCCGGAATTATTGGGCGTAAAGCGCGCGCAGGTGGTTTCTTAAGTCTGATGTGAAAGCCC
- ACGGCTCAACCGTGGAGGGTCATTGGAAACTGGGAGACTTGAGTGCAGAAGAGGAAAGTGGAATTCCATG
- TGTAGCGGTGAAATGCGTAGAGATATGGAGGAACACCAGTGGCGAAGGCGACTTTCTGGTCTGTAACTGA
- CACTGAGGCGCGAAAGCGTGGGGAGCAAACAGGATTAGATACCCTGGTAGTCCACGCCGTAAACGATGAG
- TGCTAAGTGTTAGAGGGTTTCCGCCCTTTAGTGCTGAAGTTAACGCATTAAGCACTCCGCCTGGGGAGTA

**Fig. 5:**
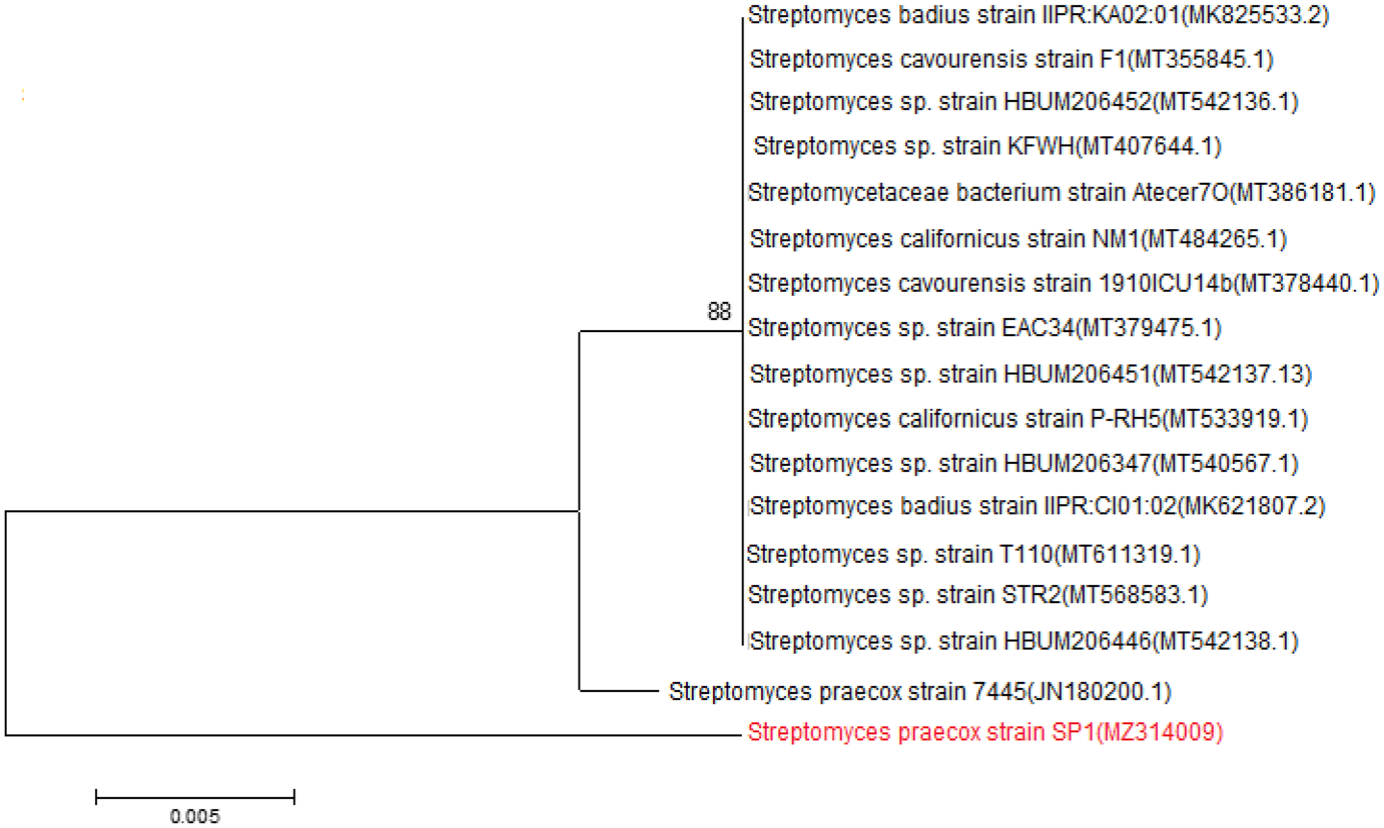
Phylogenetic tree showing the evolutionary relationship between *Streptomyces praecox* 16S rRNA nucleotide sequence genes (Strain SP1; accession number MZ314009) and the other *Streptomyces* sp. presented in the GenBank. The tree dendrogram was constructed using the neighbor-joining method using the Mega software version 6

**Fig. 6:**
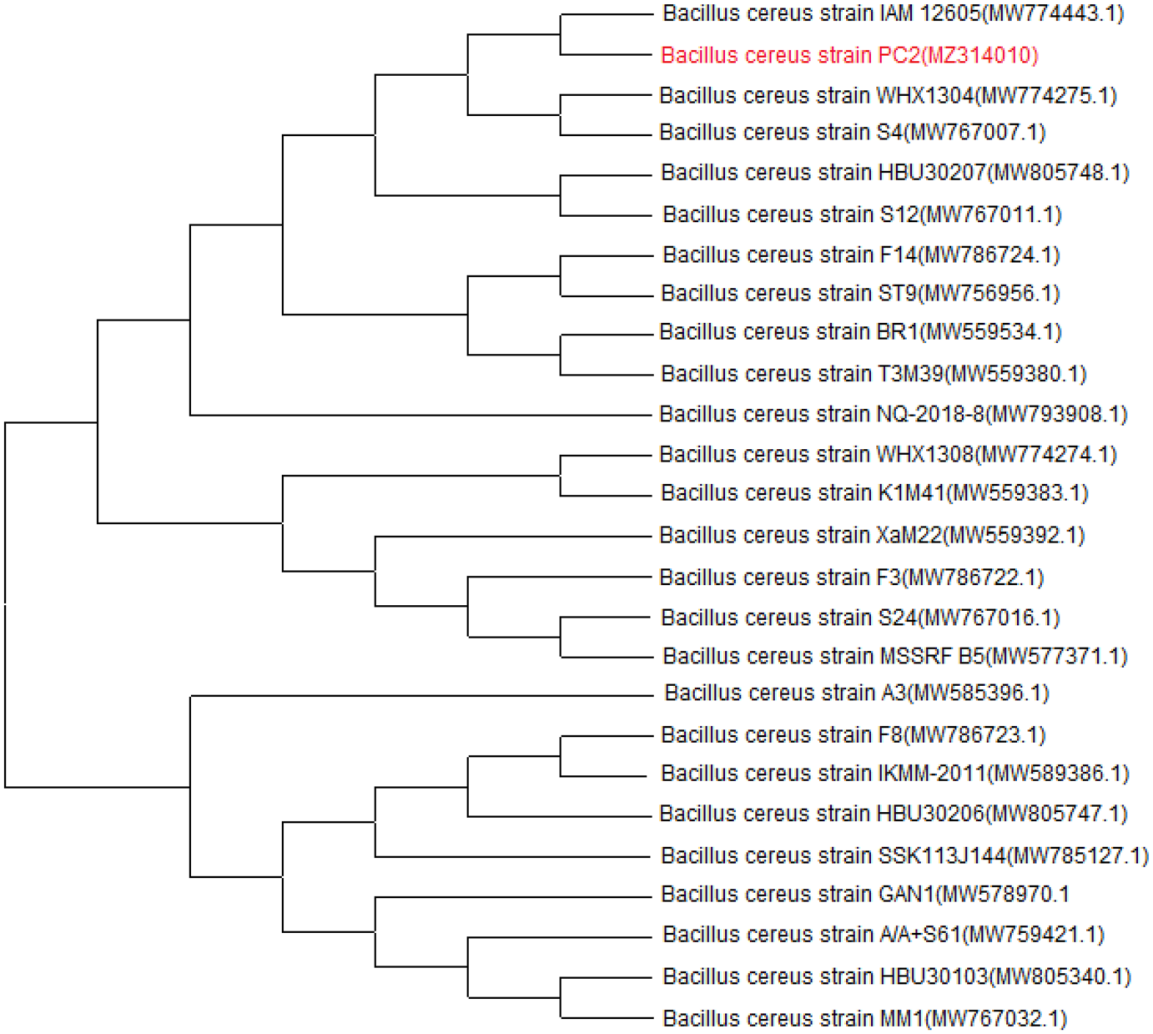
Phylogenetic tree showing the evolutionary relationship between *Bacillus cereus* 16S rRNA nucleotide sequence genes (strain PC2; accession number MZ314010) and the other *B. cereus* presented in the GenBank. The tree dendrogram was constructed using the neighbor-joining method using the Mega software version 6

The identified 16S rRNA gene sequence of *S. praecox* strain SP1 was compared with the other 16 species of *Streptomyces* in GenBank. The neighbor-joining phylogenetic tree revealed high homology between *S. praecox* strain SP1 and *S. praecox* strain 7445 (Fig. 5). Also, the phylogenetic tree of the gene sequence of *Bacillus cereus* strain PC2 with the other 25 strains of *B. cereus* strains in the GenBank revealed high homology with *B. cereus* strain IAM 12,605 (Fig. 6).

## Discussions

Indigenous microorganisms in water might define the fate of applied pesticides, undergoing degradation, transport, and adsorption/desorption processes (Kaur & Garg, 2014). The intact and degraded products of pesticides affect, significantly, these microorganisms, thus altering their microbial diversity and might cause pollution (Díaz-Cruz & Barceló, 2006). Chlorpyrifos was first introduced in 1965 by Dow Chemicals in the USA to manage a diverse number of agricultural and domestic insects (John & Shaike, 2015). It is extensively used in Egypt to control various insects and mites on a variety of field crops and residential plants (Aly et al., 2021). But it might adversely disturb ecosystems through serious environmental pollution (Cycoń et al., 2009; Liu et al., 2012). CPF was reported to cause adverse effects to pesticide applicators and farmers during its application (Farahat et al., 2010). Furthermore, pollution with CPF might affect microorganisms and non-target bees, wasps, and aquatic organisms (Jabeen et al., 2015), where it persists in neutral soil with a half-life ranging from 35 to 78 days (at 25 °C). But when it was used as a termiticide, CPF remains for 175–1576 days (Solomon et al., 2014).

The biodegradation process of CPF intermediates with the formation of 3,5,6-trichloro-2-pyridinol (TCP) as the main metabolite with greater water solubility compared to TCP. *Streptomyces* sp. strain JAAS1 was effective in degrading both CPF and TCP in contaminated sites (Abraham et al., 2013). Also, there were numerous reports on the employment of microorganisms in the detoxification of CPF from water, for example, *P. aeruginosa* (NCIM 2074) (Fulekar & Geetha, 2008) and the G1 strain of *Stenotrophomonas* sp. efficiently degraded eight OPs due to their versatile systems of genes and enzymes (Deng et al., 2015). The *B. cereus*, *B. subtilis*, *B. melitensis*, *Klebsiella* sp., *P. aeruginosa*, *P. fluorescence*, and *S. marcescens* were used CPF as main source of carbon (Lakshmi et al., 2008). In another study, *Sphingomonas* sp., *Stenotrophomonas* sp., *Bacillus* sp., *Brevundimonas* sp., and *Pseudomonas* sp. were removed from 37 to 100 mg/l/day of CPF individually. *Sphingomonas* sp. showed the greatest activity in the transformation of CPF (100 mg/l) within 24 h (Li et al., 2008). Also, *Stenotrophomonas* sp. PF32 used about 97% of 100 mg/l of CPF in 48 h as a carbon source. Moreover, the *S. maltophilia* strain MHF ENV20 degraded half of the amounts of CPF and its metabolites, trichlorophenol and diethyl thiophosphate salt in 96 h (Deng et al., 2015).

The Actinobacteria, *Streptomyces* sp. AC5 and AC7 species, effectively biodegraded CPF insecticide. The AC5 removed over 90% of CPF (50 mg/l) after 72 h. of incubation, while the AC7 strain was less effective (Briceño et al., 2012). Similarly, *Synechocystis* sp. eliminated 3.78 and 4.69 mg/l of 5 mg/l of CPF within 4 and 8 days, respectively (Singh et al., 2011). Both *Pennisetum pedicellatum* and *Stenotrophomonas maltophilia* transformed CPF to TCP and DETP and utilized them as carbon, phosphorous, and nitrogen sources (Dubey & Fulekar, 2012). Along with those, several bacterial strains were effective in degrading CPF and used it as a source of carbon, for example, *B. pumilus* (Anwar et al., 2009), *Flavobacterium* sp. (Mallick et al., 1999), *E. coli* (Richins et al., 1997), *Alcaligenes faecalis* DSP3 (Yang et al., 2005), *Klebsiella* sp. (isolated from wastewater) (Ghanem et al., 2007), *Providencia stuartii* (Bhatia, 2008), *P. aeruginosa*, and *Clavibacter michiganensis* (Subhas & Singh, 2003).

Similar to the results reported herein, there are a few fungal strains that were capable of degrading CPF. For example, *Verticillium* sp. eliminated up to 90% of CPF within 7 days at pH 7 at 35 °C (Fang et al., 2008; Yu et al., 2006). The fungal species, *Aspergillus* sp., *Penicillium* sp., *Eurotium* sp., and *Emericella* sp., degraded about 70% of CPF within 1 week and used it as a carbon and nitrogen source (Xu et al., 2007). *Cladosporium cladosporioides* Hu-01 mineralized CPF after 6 days of incubation (Chen et al., 2012). *Aspergillus terreus*-JAS1 dissociated CPF and TCP in liquid media and soil after 24 h of incubation (Silambarasan & Abraham, 2013). Compared to bacterial isolates, fungal species efficiently bioremediate CPF via a mineralization mechanism (Supreeth & Raju, 2017).

## Conclusion

Pollution of water resources with pesticides might cause serious problems. Indigenous microorganisms help lessen the adverse effects of pesticide residues through degradation. Agricultural wastewater samples were analyzed for pesticide residues, and results showed the detection of lenacil, chlorpyrifos, cypermethrin, bifenthrin, carbofuran, tolfenpyrad, oxamyl, dicloran, simetryn, sulfotep, ethofumesate, and permethrin. Chlorpyrifos insecticide was the dominant compound in wastewater samples. Indigenous bacterial and fungal species were isolated, and their ability to degrade chlorpyrifos insecticide was examined. After 1 week of incubation of isolated bacteria (D, F, and I) and fungi (A, B, E, G, H, and J) with CPF, results revealed efficiency % of removal ranging from 31.41 to 90.82%. Specifically, F (*Streptomyces praecox*), I (*Bacillus cereus*), and A (*Aspergillus terreus*) removed about 80.82, 80.93, and 75.59% of the 2000 µg/l of CPF in 1 week. Following the molecular identification of these species, they were registered in the GenBank as *Streptomyces praecox* strain SP1 and *Bacillus cereus* strain PC2 with accession Nos. MZ314009 and MZ314010, respectively.

## Data Availability

All data and materials have been presented in the manuscript.
